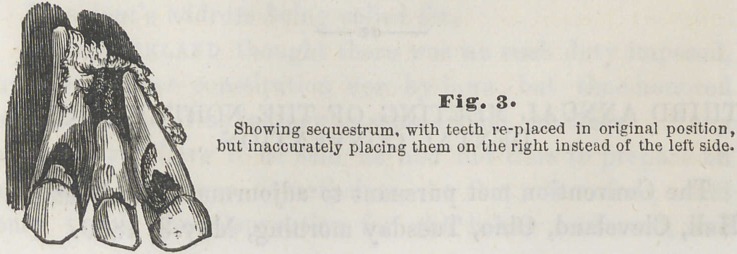# Proceedings of Societies

**Published:** 1859-06

**Authors:** 


### Proceedings of Societies.

### CINCINNATI ASSOCIATION OF DENTAL SURGEONS.

CINCINNATI, April 12, 1859.

The Association met pursuant to adjournment, at the office of Drs. Taylor & Irwin.

Present: Drs. Taylor, H. R. Smith, Taft, James, Cameron, Bonsall, Irwin, Wardle, Van Emon, and Richardson.

The minutes of the preceding meeting were read and received.

The Committee on By-Laws reported, and the Articles being taken up *seriatim*, were, after some alterations and amendments, adopted.

Drs. Bonsall and Taylor laid before the Association communications from Drs. McQuillan and J. F. Flagg, of Philadelphia, having reference to the formation of a National Dental Association, on a representative basis. The personal communications were accompanied by the following *memorial* which will sufficiently indicate the character of the movement:

MEMORIAL.

"The undersigned practitioners of dentistry, believing that a National Association of Dentists, composed of delegates from State, county, and local societies, and Dental Colleges, would be calculated to promote the best interests of the profession, respectfully suggest to the Dental Societies and Colleges throughout the country, the propriety of its electing delegates to meet in convention, at the Falls of Niagara, on the first Wednesday in August, 1859, for the purpose of forming, if the assembled delegates shall deem it expedient, a National Association, on the representative basis."

It is proposed that each Dental Society, state, county, and local, be represented in the organization by one member for every five composing such body, and that each Dental College be represented by one of its Faculty.

These communications and the accompanying memorial having been read, some expression of opinion in regard to the movement was elicited from various members present.

Dr. Bonsall remarked that he regarded the American Dental Convention as highly practical in its character, and believed it was performing services indispensable to the interests of the profession; yet he believed if it was faulty in its organization, it was in being *too democratic*, as its predecessor was too strict or *exclusive*. He did not wish to see the American Convention swallowed up in the proposed organization, but hoped it would be so constructed as to avoid either extreme.

Dr. Taft expressed some apprehension that the present movement may have its origin in a feeling of hostility to the American Dental Convention, and though favorable to it as an *independent* organization, he was opposed to any action, having in contemplation a dissolution of the existing Convention. He did not, however, believe that the latter could be so easily pushed aside, and thought that the interests of the profession would be better promoted by its continuance than its dissolution, at this time. As organized, it is, in effect, a *mass meeting* of dentists from all sections of the Union, cordially uniting their personal efforts on a common platform, without distinction or qualification, embracing every grade of professional ability and acquirement, eminently practical in its character and *progressive* in its tendencies. The creation of a Convention on the proposed basis, in lieu of the present one, invested, as it would be, with an air of aristocratic exclusiveness, he believed, would create ill-feeling and jealousy on the part of a large class of practitioners excluded from any *direct* participation in its deliberations, and thus many would be forced into an attitude of hostility, who are now heartily and efficiently coöperating with us in the advancement of practical and scientific dentistry. He thought the two organizations might exist cotemporaneously without in any way conflicting with each other, for, as he understood the objects of the proposed convention, it would probably be occupied chiefly with the adjudication or settlement of questions that could not, consistently with the functions of the American Convention, be acted upon by that body. He contemplated it in the light of a tribunal of last resort for the decision of matters that can not be authoritatively settled elsewhere.

Dr. TAYLOR, while he recognized the benefits accruing from the meetings of the American Convention, believed that one formed on a representative basis, by commanding a higher grade of acquirements, would, in many important respects, result in more permanent improvement to the profession than is likely to follow the deliberations of the present society. He believed the latter, in its practical tendencies, was efficient for good, and thought, with Dr. Taft, that the two organizations might profitably co-exist. He did not concur, however, with Dr. T., in his construction of the objects contemplated in the new organization. He was at a loss to understand what offices such an association could perform as a mere *legislative* body. He thought it would be inexpedient and unsafe to revive the experiment so effectually tried in the old National Society, of sitting in judgment on the "right of private opinion." The assumption of the right to so adjudicate, proved fatal to that association, and he presumed the profession was not more tolerant of aggression in that direction now than then.

Some further expressions of opinion were had from members on the subject, all of whom favored the movement. Members present signed the memorial, but no definite action was taken in regard to the election of delegates to represent the Association at the time and place designated.

On motion of Dr. James, a committee of three was appointed, the Corresponding Secretary to be added, to whom the matter under discussion was referred, with power to act at discretion. Committee: Drs. Taft, Irwin, Cameron, and Taylor.

The discussion of the following question, namely, "*Is dentine endowed with recuperative powers?*" being next in order, Drs. Taylor and Taft made some remarks which, in the main, were merely explanatory of its import or bearing, when, on motion, its further consideration was postponed until the next meeting of the Association, at which time Dr. Taylor consented to introduce the subject with a written essay.

Dr. H. R. Smith reported the following cases as having occurred recently in his practice.

*Necrosis of a portion of the Superior and Inferior Maxillary bones from mercurial pytalism.* Case. Child, aged two years and six months. Was recovering slowly from a severe attack of scarlatina; feeble, and greatly emaciated. When first seen, was suffering from diffused inflammation of the mouth, sponginess of the gums, excessive salivary discharges, loosening of the anterior teeth above and below, and the characteristic fetor of the breath. Several of the front teeth had already been removed. From the sockets of these and from around the adjoining teeth, purulent matter was discharging freely. In eight or ten days, nature having thrown out a barrier to a further progress of the disease, those portions of the inferior and superior maxillary bones already necrosed and loosened, were removed. The upper section embraced all that part of the jaw anterior to the deciduous molars, and extending upward, included all that portion forming the anterior floor of the nostrils, reaching up some distance upon either side of the nose. The lower section, like the upper, included all that part of the jaw anterior to the molar teeth, involving more than one-half of its entire substance.

The removal of these pieces brought with them the imperfectly formed crowns of all the permanent teeth anterior to the bicuspids above and below. The diseased condition commenced gradually subsiding with exfoliation of the necrosed portions, and the parts are now being partially repaired with granulations.

*Case of supposed Poisoning with Arsenious Acid.* Case.—Man, æt about 45 years; usually of good health and unimpair ed constitution. Called on the recommendation of his physician and desired Dr. S. to take charge of the case. On examination, the upper front teeth were found very tender to the touch, and the contiguous soft parts much swollen. The left superior lateral incisor, the patient himself had removed the day before. Pus was being discharged freely from around the central incisor, but more sparingly from the left cuspidatus. The gums around and above the left lateral and central incisors to the extent of three-fourths of an inch or more, had sloughed away, leaving the processes and maxillary bone completely denuded, and in a partially necrosed condition. The central incisor was removed.

The following is the patient's history of the manner in which he had been victimised. The lateral incisor which he had extracted, had been filled some years before, at which time the nerve had been destroyed, but the nerve canal had not been filled. The tooth gave him no trouble until recently, when it began to ache, and became somewhat tender to the touch. He was recommended to Dr. H. C. J. Richards, a dentist of this city, who proposed to remove the filling and *explore*, which was accordingly done. He at once pronounced the nerve alive, and arming himself with a broach, penetrated the nerve cavity, thrusting the instrument repeatedly through and beyond the formina, and lascerating the delicate and sensitive tissues, until the patient, tortured beyond endurance, compelled him to desist. Concluding that inasmuch as *sensibility* remained in spite of his heroic attempts to annihilate the offending organ, the learned Doctor (?) concluded to poison the life out of it. To the wounded and already inflamed tissues, he accordingly applied the unmixed arsenious acid, (infered from description of it as a *white* powder,) and the patient, (formerly a druggist,) estimates the quantity to have been about one grain. He was then dismissed, with instructions to return the following day. In the meantime, all the former symptoms, as might be supposed, were greatly aggavated. Active periodontitis rapidly supervened, with tumefaction of the gums and face, accompanied with excessive pain and loosening of the front teeth. These local disturbances were followed in a short time with unmistakable evidences of arsenical poisoning of the whole system. The patient, for the next four days, was confined, during the greater part of the time, to his bed, suffering severely from pains throughout the body, constant nausea, with frequent attempts at vomiting, physical prostration and mental dejection. He contrived, however, to leave his room, and called again upon his dentist, who, after examining his condition, assured the patient he was *doing very well*, and *re-applied the white powder!* This closed his professional supervision of the case, for the patient, having suffered a few days longer all the consequences of this unmitigated and shameless quackery, was finally recommended to Dr. Smith, in the condition already mentioned.

The treatment employed in this case by Dr. S., consisted chiefly in the free use of the nitrate of silver, tonic, and astringent washes, and such detergent lotions as seemed best calculated to promote cleanliness of the parts. During the progress of treatment, detached portions of necrosed bone, were, from time to time, removed, and in about ten days, in the attempt to remove a projecting portion of one of the processes, the entire sequestrum came away, bringing with it the left canine tooth. The mass of dead bone comprised portions of remaining processes, but consisted principally of the spongy portions of the jaw bone proper, extending several lines above the apices of the teeth that had been removed, namely, the left superior central, and lateral incisors, and left canine. The parts involved, are being restored by

**Fig. 3.**

Showing sequestrum, with teeth re-placed in original position, but inaccurately placing them on the right instead of the left side.

healthy granulations, but the patient has unqestionably suffered serious, and to some extent, permanent mutilation.

*Death and permanent discoloration of the Superior Central Incisors.* Case. Boy, æt twelve years, son of the gentleman whose case has just been detailed. Was under treatment at the same time and by the same dentist. The application of a *white powder* in this case also, was made to the superior central incisors, with a view to destroy the nerves. Three applications were made in as many days. Results: destruction of the nerves, active periodontitis, swelling of the gums and face, loosening of the teeth, discharge of pus from underneath the gums, necrosis of a portion of the processes between the incisor teeth, and a marked and permanent discoloration of the latter. These conditions were promptly treated by Dr. S., who succeeded in so far subduing the inflammation and controlling the discharge as to enable him to determine upon their retention in the mouth for the present, and if practicable, until the jaw shall have acquired its full expansion, when the teeth will have to be removed, as they are likely to remain permanently blackened.

The relation of the above cases drew forth from members present, expressions of unqualified censure, and it was unanimously,

*Resolved*, That the Corresponding Secretary be instructed to inform Dr. Richards that this Association is aware of his mal-practice in the use of arsenic, alias *white powder.*

On motion, the Association adjourned, to meet at the office of Dr. Cameron on the second Tuesday of May, 1859.

J. Richardson, *Sec'y.*

### THIRD ANNUAL MEETING OF THE NORTHERN OHIO DENTAL CONVENTION.

The Convention met pursuant to adjournment, at Fremont Hall, Cleveland, Ohio, Tuesday morning, May 3, 1859.

*Members Present.*—Benj. Strickland, C. R. Butler, B. F. Robinson, F. S. Slosson, W. H. Atkinson, M. J. Dickerson, B. A. Halliwell, E. G. Burger, J. G. Moore, B. F. Spelman, C. Palmer, M. Palmer, —— Burroughs, —— Iddings, S. R. Huntington, E. J. Way, —— Siddall, —— Storrs, C. P. Baily, —— Wilson, R. Varney, Geo. Cole, G. Longsdorff, —— Crowell, —— Guyld, —— Daniels.

The President, Dr. Benjamin Strickland, in the Chair. Minutes of last meeting read and adopted.

On call of Committee on Dental Reports, Dr. Robinson, chairman, asked further time. Report on Constitution and By-Laws being called for, committee not being ready to report, the subject was deferred until Wednesday morning.

The Executive Committee reported the order of business, as follows, viz:

SUBJECTS FOR DISCUSSION.

1. Best means of correcting irregularities of the teeth.
2. Treatment of Exposed Nerves.
3. Treatment of Alveolar Abscess.
4. Filling Teeth and Fangs.
5. Mechanical Dentistry.
6. Miscellaneous.

B. F. Robinson,<br>W. P. Horton,<br>B. F. Spelman, } Committee.

On motion, adjourned to meet a 2 o'clock P. M.

FIRST DAY—AFTERNOON SESSION.

Met according to adjournment. Minutes read and adopted.

President's address being called for,

Dr. Strickland thought there was no such duty imposed, having neither constitution nor by-laws, but time-honored custom makes it obligatory to some extent, he knew. They would not believe if he said he had not time to prepare an address. Such was, nevertheless, the fact; therefore, could only thank the Convention for the honor conferred, and would direct their attention to one Bright Spot, where he hoped to meet all here present, besides many hundreds of others, joined in the same holy cause from East, West, North and South, who, as brothers, earnest in the elevation of our beloved dental science, would meet at Niagara next August.

The remarks of the President were received with hearty applause.

On motion, it was unanimously

*Resolved*, That the widest scope be given to the discussion.

FIRST SUBJECT—*Best Means of Correcting Irregularities of the Teeth.*

Dr. ATKINSON said that he was sorry that we knew so little what to do in such cases. Indeed, there are few who know anything about it. Irregularity may best be prevented by living nearest to the physiological laws, for it is certain, that if we always live according to correct laws, nature will always give a perfect organization. It is impossible to discuss this subject in full, unless it is done in pieces, therefore, I don't know where to begin or where to stop. We have to do with vital principles and vital actions. We can accomplish the best end only by operating as if looking forward to a time when we will no longer be needed as dentists. We must, therefore, take cases as they come, and determine, in children, *when* to extract, so as to avoid irregularity. Neither parent, patient, or operator have given the subject proper attention, either from indolence or incompetency. We occupy a high responsibility. If the dentist, as a member of the human family, will endeavor to attain the highest point of Eutopian perfection, then there will soon be no need of dentists or physicians.

Dr. C. PALMER said he had lived among the Indians and practiced medicine among them, and never found decayed or aching teeth, so as to require extraction; but when they become civilized, their teeth decay rapidly. Never knew of a case of irregularity among the Indians.

Dr. ATKINSON replied, that this was no offset to his remarks.

Dr. SPELMAN said, dentists may theorize as much as they please, but patients will not take advice nor a single step toward an initiary course to prevent irregularity; therefore, we must take cases as they come, and treat cases as we find them. When patients demand to have teeth extracted, to have a handsome set of artificial teeth—with utter disregard of the value of these organs—it is, useless to lay down any rules. Might give his mode of practice, but no doubt, others are familiar with the same process.

Dr. PALMER inquired whether, in cases of contracted arches — teeth standing behind each other, should any be extracted?

Dr. ATKINSON replied, "Never!"

Dr. SPELMAN has, in cases of irregularity of seven year molars, moved every tooth in the mouth and brought all within the arch. Takes a soft iron wire and attaches it to the molar, or any other tooth, passing it around the front tooth, or teeth, so as to bring the pressure in the proper direction. A case, in which the distance between the molar teeth was only 6-8th of an inch across—nasal passage almost closed. Patient much alarmed, as an attack of quinsoy, or anything of the kind, would prove fatal. The lateral incisors were directly behind the centrals, and the centrals so close together that a knife could not be passed between them. Concluded that by expanding the jaw, the cuspidata would just come to the proper place. By means of wire and ligatures, as described, the jaw was expanded seven-eighths of an inch more than when commenced. Began Jan. 1st, 1859, and expected to have casts ready to exhibit before this Convention, but was disappointed—there were four weeks of intermission on account of cancerous sore mouth. They are now almost all in regular arch.

Dr. WAY wished to know when it is best to regulate, or extract teeth for the purpose of regulating?

Dr. Atkinson replied at some length. All we want is traction, applied at the proper time, in the proper way, and for the proper length of time. *Contraction* never occurs without the loss of substance. It is simply a want of development. Prefers to let the fang become complete, or nearly complete, before commencing traction. Then move steadily and persistently in the direction desired, until the proper relation is attained, then *retain* in position until they become permanently fixed, which will occupy from six weeks to a year; being exceedingly careful to cleanse the mouth frequently to avoid the generation of acids, and consequent solution of tooth substance. The reason for requiring the teeth to be nearly complete, is found in the increased length of the lever, and the avoidance of curvature in the fangs, by which means we can increase or diminish the aveolar arch; but would prefer such cases as require increasing rather than those that require diminishing. Very much can be effected by gentle and delicate means, if the patient applies sufficiently early, even before the completion of the fangs, to do which successfully, each individual case will be its own best indication, to the intelligent, appreciative dentist, many operators of equal merit preferring different modes of applying the force.

Dr. Way wanted to know if extracting teeth too soon is not a cause of contraction, or, as Dr. Atkinson remarked, want of development?

Dr. Slosson would prefer patients at 16 or over. No doubt when teeth are prematurely extracted the arch is contracted, or fails to *expand*. The natural arch is lost by the extraction of canines, which he always refuses to do. Can not throw the arch backward, except the wisdom teeth are extracted; usually there is a narrowness of the jaw. Mentions case of a girl who could not close the jaw over. Commenced by taking out a decayed molar, and pushing back the bicuspid on opposite side. He pushed back until the space was filled. Still the jaw stuck out pointed. He then used ligatures, until now you would not know the lady; aged 16, and an attractive match for some young man.

Dr. ATKINSON has heard favorably of the case, since treating, and is satisfied that it has improved since, by the elasticity of the muscles and process.

Dr. SLOSSON reports another case of a different character, being a slight separation between the incisors; process soft. Cautioned patient. In after examination found it had separated much more, leaving a space as wide as the central incisor, and on probing found a separation in the process of upper jaw, and still widening, and the process absorbing so that the point of the tang could be punctured with a probe. It is both a separation and absorption—absorption around the centrals and laterals, and a separation between the incisors.

Dr. SPELMAN says, as far as the osseous structure is concerned, the case appears like cleft palate. Would continue to attempt to regulate at any age from 16 to 35. The length of time required is different in different cases.

Dr. BUTLER related a case having been regulated, and in six or eight months the teeth returned to the irregular position in which they were before. Thinks the reason was that owing to the age of the patient, it required greater time in keeping the teeth in a regular position, in order to make them remain.

Another case aged 13 or 14 years. Found, on examining, the second bicuspid was decayed. Concluded if it was removed the arch would become one-sided. Concluded, therefore, to remove that and the corresponding tooth on the other side. The teeth took proper position, and the arch is now beautifully regular.

Dr. SLOSSON reports case of a girl of 5 years of age, who, when she closed her jaw, there was a hole as large as a pipe stem and seemed as if bored out. Thought she must have been sucking her thumb.

Dr. BUTLER reports a similiar case. Done nothing, but gave a good lecture, and this prevented the evil.

Dr. Moore thinks the deciduous teeth are for no other purpose than to guide the permanent set. They want to be attended to, and kept clean and free from disease, regulating by pressure of finger, then the second set will come regularly and follow the deciduous. The fangs of the deciduous absorbing as the permanent make progress. God made the deciduous teeth only to develop the jaw and make room for the permanent. Case of teeth produced after the age of 35.

Dr. Spelman said, not more than a week since a gentleman sitting in his office remarked to another, (a patient,) if you was as smart as I, you would have another set; that within five years he had been supplied with another, or third set. On examination, I discovered that by direct occlusion the teeth had so worn down that it appeared like two rows of teeth.

Dr. Way thinks by early extraction of deciduous teeth, the permanent are frequently smaller than they otherwise would be.

Dr. Bailey saw a case in New York, of a gentleman who had not received his permanent set until the age of 47; had two of the teeth attached to each other, and he also stated that he had two extracted on the opposite side, which were attached in a similar way. Some claimed it was his third set, but he acknowledged his incredulity.

Dr. Butler asked about teeth standing precisely edgewise.

Dr. Slosson thought they might be regulated by india rubber ligatures.

Dr. Spelman had not much to say, but hoped to draw out others. When Dr. Slosson spoke of his case it immediately suggested itself to his mind how he would treat. He would adopt means to confine the jaw, at least, so that it could spread no farther, and would then endeavor to bring them together. Nature abhors a vacuum, and fills up space either with soft tissue or with cartilage, which, by pressure, would absorb. If this would not do, would resort to the knife, and with sharp chissel cut the edges of the bone and spring them together, so that nature could throw out ossific matter, to form a reunion.

Dr. PALMER thought it must be absorption, and if such was the case they would not unite when brought together.

Dr. ATKINSON thinks this legitimately comes under the head of alveolar abscess. There are two conditions under which these cases present themselves; either the system has been mercurialized, or else is charged with that delicate disease called syphilis. In either case, constitutional treatment must be resorted to, and continued until ossific matter is thrown out and a union formed.

Dr. WILSON, of Rochester, N. Y., said: I am here as a stranger, and did not expect to say anything, but came to listen. Speaking of irregularities, will mention case of a young man, 19 years of age, with whom nothing was supposed to be the matter. Irregularity of incisors above and below; had uniform cuspids. The incisors stood out so much that there seemed to be no room for them, and concluded must remove tush to make room for them, and did remove bicuspids. Kept in charge, and has ever since been all right.

In another case of irregularity, I used a cord, or ligature, passed over and around the teeth, so as to bring the pressure in the right direction; tightening and changing until they were perfectly regular. In regard to a third set, never seen but one case, and that, an old lady 75 years of age, who had lost all but three teeth, who declared she had a new tooth appear where none had been for years.

Dr. HORTON does not think he knows anything more than others, but each have patients and cases presented under different circumstances and conditions. Mentions a case of a young man of powerful frame, robust, and healthy, who had one tooth entirely inside the arch. He wanted to have it extracted, but yet, regretted the necessity. Therefore, I concluded to try to regulate. Took impression of lower jaw, struck a plate, and built an inclined plane so that he could not close the mouth without striking the tooth first. Recently heard from him, and the tooth is now in the proper position, sound and healthy. His age is 22.

*Another.*—Incisors lopped. Applied a ligature, and lever, or silver spring, until the proper position was attained.

*Another.*—The case of a lady, where an incisor *projected.* Applied India rubber, silver spring, and bound with floss silk, with injunction to return; but patient removed ligature, and the case did not entirely succeed.

Several other cases of irregularities were related of different characters, which were treated by similar means, and with the happiest success.

Dr. HORTON here remarked upon the class of patients in which irregularities occur. It is not among the children of the Irish in the shanties, nor the Germans, but generally among the wealthy. A case of a family of healthy, robust people who, raised in poverty, suddenly became wealthy. A child of theirs had very irregular teeth. The inference drawn was, that it occurred from the food and cultivation of the child.

Dr. WILSON says sweetmeats and high living does not do all the evil, but tends to produce decay and destroy the temporary teeth, which produces contraction of the jaw, and, of course irregularity of second dentition. For want of room, therefore, fills temporary teeth, and tries to save them as long as possible.

Dr. STRICKLAND—Have been so much interested in the discussion that I have scarcely thought of saying anything myself. Treat irregularities differently from many others. It is by the expression of our views and illustrations of cases, that we come at correct ideas. I make it a rule to commence as early as practicable, and apply pressure as rigorously possible, regardless of the crooking of fangs, as expressed by Dr. Atkinson. Sometimes we find dead fangs. In such cases it should be removed as early as possible. A dead fang, being incapable of being absorbed, should be removed at once. Adopts different methods of regulating; for such purposes I generally use a struck plate, (atmospheric,)

to which any appliance can be attached. To move the tooth out or in, screw on ligatures. Prefer to commence before patients have attained their growth; would not attack such cases except important ones, as it would take much time in an adult.

SECOND SUBJECT — *Treatment of Exposed Nerves.*

Dr. SPILMAN says: Have spoken on that subject in this Convention. In general practice treats nerves severely. Kills them, yet does not think it the best practice. If in a city, would do otherwise; but patients living 10, 12, or 15 miles away, will not come in to have the application renewed at proper time. When he does treat, it is with creosote and tannin, (suggested by Dr. Geo. Watt,) and covers with gutta percha to prevent conducting of heat and cold, but really has nothing new.

Dr. PALMER, of Warren, says: Have not been here at a previous meeting. When much tenderness, indicating the nerve is nearly exposed, uses a burnisher rubbed briskly over the surface, which removes the sensitiveness.

Dr. BAILEY, of Cuyahoga Falls, has nothing new, but wishes to correct a mis-print. Believes he stated that he generally used for destroyiug teeth, creosote, arsenic, and morphine. Arsenic, 10 gr., morphine, 15 gr., and creosote. Has met with such general success, that he has sought nothing better. Uses the same for sensitive dentine, four and six hours, then applies tinct. Rhatany. Sometimes uses other astringents. Has sometimes a case of soreness—does meet with failures, but of such rare occurrence in proportion to the number of cases treated, that he thinks that it is the imprudence of the patient in taking cold, or otherwise. Generally retains mixture five or six hours; excavates thoroughly down as far as the *live* part of the *nerve*, fills without capping, so that when swelling occurs, there is room for the expansion of the pulp. Wants nothing better. Fills in about four days after removing the nerve medicine, treating in the mean time, with tinct. Rhatany.

Dr. SPELMAN differs from Dr. Bailey. When he permits arsenic to remain in a tooth five or six hours, (he uses about the same preparation,) he finds the nerve entirely destroyed, and his first step is to probe to the end of the fang, and finding no soreness, except it occurs from excessive use of the arsenic, therefore, can not understand how the nerve can be killed only *part way.* Understands that the nerve is accompanied by a vein and artery which, according to his views, is inconsistent with the partial killing of the pulp. Yet, would not say that it is not so. The Doctor *may* be correct; the idea is new, but we are constantly hearing of new things, and when they come on good authority, as this does, we are bound to believe it, however imperfectly we may be able to comprehend it. If he fails in one case out of four, he does not consider himself successful, and would abandon the profession if he could not do better.

Dr. BAILEY inquired if Dr. S. would consider 47 cases out of 50, a success?

Dr. S. replied he certainly would.

Dr. BAILEY continued. If any gentleman will follow the directions here given, he will undoubtedly meet with that ratio of success. Such having been his practice, and such the result.

Dr. ATKINSON expressed himself much interested. Dr. Slosson and himself (when practicing together,) have frequently seen the phenomena of nerves bleeding, without sensibility. When we can not, by our best treatment, bring about resolution, or new granulations, then extirpate and fill hermetically; it is a mere matter of *time*, and patient, persistent control of the case. In cases where, from any cause, we can not have the necessary time, obtuned the sensibility with cobalt and creosote; extirpate and fill. To be successful, let every man behave honestly at heart—it is necessary to attend to some detail—and lest we should leave some fragment of the soft tissue in the smallest parts of the canal, my method is to saturate the canal well with creosote, preferring an excess to a deficiency, leaving a small pellet of cotton in or near the apex of the fang; after which fill in in the usual manner.

Take a *case.* A patient presents himself with a nerve exposed, and the surface suppurating. Cleanse and apply a pledget of cotton, wet with pure creosote, to the secreting surface. This will arrest the secretion of pus, if not allowed to remain long enough to destroy healthy tissue, thereby causing a secondary slough. Wash out with a strong solution of tannin; cap and fill. The true indication of a tooth being in a fit condition to fill, after being treated in this manner, is known by its not bleeding when syringed with warm water, or bland fluids, in other words, when a perfect cicatrix had formed. I can conceive of this slough involving all that portion of pulp in the pulp chamber, leaving only pulp of fang: thus explaining and accounting for the cases reported by Drs. Slosson and Bailey, as successes. Nevertheless, I do not think it requires more acumen than is generally possessed by members of the profession, to ascertain *just the point* when a tooth, under such circumstances, can be safely filled, and until the basal sciences are more generally studied and understood, I would advise extirpation in all cases at all doubtful. At the same time expressing my firm conviction and confident hope, that the time is not far distant when this matter will be understood and treated as it should be; I mean to say when we shall succeed by rule and fail by chance, and not, as heretofore, fail by rule and succeed by chance.

Dr. Spelman declared himself lost. With his theories considerably staggered, feels lame, but still disposed to fight. Confesses that Dr. Atkinson knows more about the human system than himself, or probably any one present; would, therefore, ask a question, viz: Does the nerve *terminate* in the tooth?

Dr. Atkinson replied: The termination of the nerves can only be seen by microscopic observation, and it is doubtful whether we yet know by our best glasses, the absolute termination of the nerve cord. The pulp is composed of celular tissue, blood vessels, and nerves, constituting a truly animal force of life. Study the minute anatomy and the ground-work is laid.

Dr. SPELMAN can not understand how, if a nerve with the accompanying artery and vein, are cut off in the tooth, and the artery pouring out blood, how can it heal up? By inflammation, it would be out of the fang.

Dr. WAY said different articles seem to operate differently. Arsenic is absorbed into the circulation; creosote kills only so far as it comes in contact. In practice, has found chloride of zinc the best for sesitive dentine.

Dr. PALMER remarked that escharotics are different from each other in their operation. Some penetrate more rapidly and deeply than others. Arsenic penetrates and kills the nerve. It also penetrates the bony substance of the tooth. I have abandoned and promised never to use it again, and thus far have kept my word. In conversation with J. D. White, the latter recommended cobalt, and I have used it ever since, it does not so rapidly attack the bony part of the tooth. Prefer to extirpate when *practical.*

A gentleman was now called forward to exhibit his teeth. He stated that he lost the permanent set between the age of 10 and 18. At the age of 19, had a severe attack of typhus fever. At 19, had another attack, and at 21 had not a tooth in his head. At the age of 23, three upper incisors made their appearance, and in rapid succession, one followed another, until the entire arch in both jaws was restored with teeth, fine, healthy, and well developed.

Adjourned to meet at 7 o'clock, P. M.

### EVENING SESSION.

Dr. BAILEY reported a case where arsenic was applied in 18 cavities in the same mouth, at one time, and with perfect success.

Dr. ROBINSON would be glad to find some way by which he could save the nerve alive in the teeth, but confessed his want of ability so to do. Excavates and exposes the pulp; then carefully punctures, to make it bleed; then applies cobalt and creosote, and allows it to remain from four to six hours, and generally finds the nerve dead, which he removes at once; then cleanse and fills to the point of fang. Cuts until the hole is large enough to reach the point of fang, when there is uncertainty of reaching to the point.

Dr. MOORE had not been successful in his treatment of nerves. Almost came to the conclusion to abandon the attempt, but recently adopted Dr. Robinson's mode, which produced much pain. Would now rather go the whole figure, viz: *Extract the tooth, cleanse out the fangs and cavity, and replace in the socket.* Has now several cases in the city with teeth in the mouth that have been *extracted and replaced*, and are as solid and as useful as they were before they were decayed. Treats nerves as an " open enemy," and when he does treat them, he does it with carbonic acid, with which he destroys nerves or allays sensibility. Much rather at any time, extract the tooth, plug, and replace it; having the utmost confidence of success. In all, has treated about one dozen cases. Others have treated many more.

Dr. SPELMAN thinks it would be useless to fill fangs. Having them out of the mouth, you might as well drive in a tack. This is a new era in dentistry, and probably it would be well to open an office, send out circulars, and have patients *send in their teeth by letter*, fill, and return them. Knows a dentist who claims to kill all the nerves, which, however, are followed by alveolar abscess. This is not the right course for dentists. We should take a higher stand.

Here a few personalities were indulged in, which were promptly suppressed by the President. Order being restored, the discussion was amicably resumed.

Dr. SLOSSON reported a case of a gentleman in this city, having a tooth which was extracted and filled seven years ago, and is now worn with comfort and satisfaction. Thinks it can be done, although he has not done it himself with any success. He tried some cases, one of which would have succeeded if the patient had been patient. Would now refer to his own mode of treating nerves. He treated by the same mode as described at last meeting. That was, cut off the nerve as high up as is consistent, and treat with creosote and tannin. Believes in capping, and puts in the cap "wrong side up," as Dr. Atkinson says, and the more he does it, the more he is convinced it is the best way. Files the top of the cap flat, so as to make the edges thin, so that it models down, and to the tooth with, and like the foil. Has used cobalt, and filled to point of fang, when he could, and has succeeded better than he expected. But the greatest success is in capping. Generally dips the first piece of foil in creosote, and when the filling is solid, it seems that the creosote has all worked clear out to the surface. Sometimes wets the cavity with creosote and wipes it out before filling.

Dr. Siddell reports case of a lady who had all the teeth on one side decayed, the other being good. Wisdom and molar teeth on one side had the pulp exposed; could not see into the pulp cavity without a glass. Removed pulp down to the fang, and filled with amalgam. This occurred six months ago, and the case is doing well.

Also knew a case of a little school girl who fell and knocked out a front tooth, which she carried home in her pocket. Saw her several years after, and it had been replaced and had become so firmly fixed and healthy, that he could not discover the difference between that and the others.

Related two cases of teeth being extracted and replaced, after having been filled which were successful.

Dr. Atkinson, having reported his method so often, thinks they should believe it, if only to accommodate him. Had no experience in extracting teeth, filling, and replacing them; but has, in a few cases, extracted a sound tooth by mistake, and replaced it, and in all cases they have become again attached, and, to all appearances, sound and healthy.

Dr. Robinson says the first tooth he ever extracted in Ohio, was a sound tooth, by mistake. Cut around the right one, and took out the wrong one. It was a cuspid which he replaced, and extracted the bicuspid. The cuspid is now sound and healthy.

Dr. Strickland has no doubt all are tired, but still must say a word; and if anything is said conflicting with others, it must not be considered personal. Our remarks are public property. Have had some experience with arsenic. Commenced twenty years ago; it is a powerful escharotic, and a powerful irritant. We use creosote with it because creosote paralyizes the nerve, while the arsenic operates as an escharotic, and destroys the nerve. Some use morphine, in addition, but we do not think anything is gained. When he wants to preserve vitality, never uses arsenic, because it can not be done with safety, for when once introduced no one can tell when it will stop. Veins and arteries both accompanying the nerve into the tooth, will be most likely to take up the arsenic. Would never feel confident of success, or sustaining vitality in a tooth, after medication by arsenic. Would prefer any way that would be justified in the pain; electricity, or even the naked instrument, if the patient would submit. Arsenic is colorless, therefore, it is not that which discolors the tooth, but the destruction, or stoppage of the circulation, or some chemical change, Cautions young men against the free use of arsènic. Cobalt differs from arsenic only in activity.

Dr. Atkinson says it is simply the disintegration and breaking down of the tubuli.

Here the discussion took a conversational turn, highly interesting and instructive, in the relation of cases, etc.

Third Subject — *Treatment of Alveolar Abscess.*

Dr. Slosson commenced this subject by relating the case of a lady with an abscess, which he probed through to the root; treated with creosote, and filled to end of fang. Heard from the lady a few days ago, when all was sound and healthy.

Since last Convention had some cases of diseased antrum; from teeth decayed. Extracted teeth, and on one of the roots there was a large sack. The patient found blood pass through the nose. Probed to the floor of the eye and applied creosote on a pledget of cotton, first washing with a syringe, when it soon got well.

Another case, where pressure on the lip would produce a discharge, which was very offensive. Probed into the antrum, opened up the cavity, and removed a piece of diseased bone, and injected a solution of nitc. silver, creosote, etc.

Dr. Spelman, to avoid personalities, will proceed instead of following. Hopes Dr. Palmer will not so consider him now, as he expects to speak of him. Case. Last fall a lady who had a considerable amount of work done, and had, as she thought, exhausted her purse, had a tooth with an ulcerated fang, which he agreed to do for $7, with tin. Dr. Sawyer suggested to him that Dr. Palmer performed such operations without treatment. This gave him courage. He drilled to the end of the fang, and filled with the tin foil. Has seen the lady frequently, and recently. The abscess has entirely healed. The reason is, doubtless, that the cavity in the tooth is the receptacle for the pus emanating, which continues and extends irritation. This being closed, it has no place to lodge this irritating substance. Has treated several cases since in the same way, with varied success. Change of temperature is the principal cause of abscess. When it does occur, the deleterious effects of the matter must affect the whole economy of the tooth.

Dr. Moore wanted to know if abscess could occur without decay of tooth?

Dr. Strong had treated an abscess for two years, without success. The patient could not open the mouth. Extracted the tooth (a wisdom) and the abscess immediately healed, and the mouth could be opened with facility.

Dr. SIDDELL reports a case of abscess which was treated constitutionally by a homœopathist, with perfect success.

Another, where the tooth was very troublesome and soreness at point of fang. Removed filling, and drilled through. Destroyed nerve, and filled to apex of tooth. The tooth got well.

Dr. PALMER: First thing he does is to cut into the tooth until you can reach the pulp, continuing to remove the decay of the fang until it is white, then fill with gold, as perfectly as possible, and with as little delay, as all time lost increases the difficulty. Has treated several cases in this way, with perfect success. It must be done well and perfectly. Instruments must be sharp. Always fills fangs when he expects to set a pivot tooth. Wants his instruments made of tough steel, soft and delicate, so as to pass up well; to be round on one side and flat on the other, with instrument bent, and point as hard as it can be made to cut; all the rest soft. This is accomplished as follows. As far as you want it hard is nearly the shape of a spade. First harden, then draw temper out of all but point; it is a delicate matter, but should be brought to a heat just right, and not be withdrawn, but hold the point with a pair of large cold plyers, or tongs, and draw temper from balance.

Dr. BAILEY, of Cuyahoga Falls, says a gentleman applied to him with a molar tooth, which was painful and troublesome. It was much discolored. He agreed to treat it if patient would attend to directions. Removed filling of tin, it then discharged offensive matter. Then injected into it tinct. of rhatany. In a few days the patient was called to Chicago, and on his return found case much improved. Repeated the injection of tinct. of rhatany. Patient was again called away, and remained about three weeks. Continued the treatment of rhatany for seven or eight days, and obtained, apparently, a perfectly healthy condition. Excavated freely, and cleansed to point of fang; drilled through to make it larger, packed cotton in front of cavity, filled fang, and afterward the tooth, with perfect success, with which the patient was much pleased, besides paying well for it.

Dr. ATKINSON agrees as begun by Dr. Spelman and ended by Dr. Palmer. When you find a tooth which, under pressure of finger, or appulse of mallet, does not give pain, the sooner it is filled the better; but if painful, "look out," you have something more than "child's play." Know your work and get at the bottom. It requires more than a novice to diagnose properly. Creosote is not "an enemy." You had best cut several cases, than fail to cut where you should. Periostial inflammation is the great trouble. Had a case of a molar tooth; treated with creosote. Filled two fangs with comfort; began on the third, when symptoms of pain were apparent; stopped. Treated a week and filled, and it was a success. The rule is, to cut to point of fang, and introduce pledgets saturated in creosote, so as not to distend uncomfortably. Some prefer one article, some another. He preferred creosote, *pure* creosote, not pyroligneous acid.

Adjourned to meet at 8 o'clock to-morrow morning.

SECOND DAY—MORNING SESSION.

Met at 8 o'clock, pursuant to adjournment.

FOURTH SUBJECT—*Filling Teeth and Fangs.*

Dr. SLOSSON thought this subject had been discussed along with the third subject. He is in the habit of filling fangs; frequently hear people talk of filling to the *extremity* of the fang, but finds it very difficult to get to the *end;* the *nearer* we get there, the better.

Dr. SPELMAN wished to know whether, in drilling out the fang, the Doctor had ever passed through into the investing membrane?

Dr. SLOSSON replied that he had done so in a few instances, and that he had made great efforts to *save* teeth, that, with him, being the chief end, while with many, the teeth would be extracted without an effort to save them. Filled a great many fangs; many of them lasted six, eight, and even ten years, while others lasted only one or two years. Other operators would have extracted and obtained more credit. Filled sometimes over exposed nerves, and seen others do so. It requires more skill to save a tooth than to extract one, and this should be the aim of every practitioner. Often extract teeth when they could be saved, if the patient would give the time and attention necessary to have the case properly treated, and pay for it. The right way is to *save.* When there is much inflammation, treats by counter-irritation; if this fails, then drills through and excavates the fang, etc.

Dr. Strickland arose to make a correction. His ground is that the profession should be sure they are right, then go ahead. Believes his efforts in his way, have been as great as those of Dr. Slosson; but they go about the work in a different manner. He would remove all the soft tissue and fill to the end of the fang, where it is possible so to do. Never extracts when he thinks it *possible* to save the tooth. The chief difference between him and Dr. Slosson is, that he *removes* what Dr. Slosson *covers up.* Suppose you apply poison, which destroys one-half, and the poison is absorbed into the other; it is possible, even probable, that the poison is absorbed, and with a good filling, is shut up, (having no chance for the escape of gases,) to go on and produce further decomposition. If we could apply an escharotic to destroy only what we wish to remove, it would be well; but too much caution can not be observed. If we can remove the soft structure and fill solidly with gold, it is better than to cover up and enclose poisoned dentine.

Dr. Slosson was pleased to see he had accomplished more than he had expected, in "waking up" the old gentleman. If a nerve can be retained in a healthy condition, supplying vitality to the tooth, it is a better filling than any gold filling he, or any man, can put there. The nerve may be punctured and bled, and yet be recuperated so as to restore the tooth to vitality. Thinks there is a great difference between the effects of cobalt and arsenic; prefers the former because it does not travel so fast. If allowed to remain in the tooth just long enough to do the work, and no longer, then excavate freely to the extent that the poison has gone, put in a perfectly solid filling of gold, and the very best results may be expected.

Dr. SPELMAN regrets that we have not a full Convention, as this is the first time that we have had anything like a discussion. In regard to filling fangs, he has had some experience. As to filling *to the apex*, must confess he had not succeeded in bicuspids with two fangs, narrow and crooked; in using the smallest drill, has passed through at the bend, into the soft tissue, through the process, so that he could see the motion of the drill in the gums. When he has gone as far as he conveniently can, he generally stops; finds it difficult in molars, where the roots are bent or compressed, even when using drills almost as small as a cambric needle; even then it is sometimes impossible to go to the *end* of the fang. But in some cases he has passed entirely through the apex. Don't think it important in all cases that the filling should extend to the apex; would rather stop before quite reaching, than to pass through and create inflammation and disease there, where it is difficult to treat. Uses arsenic, and after the application generally finds the nerve entirely dead clear to the apex. The most of his patients came from a distance. Applies arsenic, morphine, and creosote; directs to let it remain five or six hours; remove and wash the mouth well, and return in 24 or 48 hours. Then finds some sensibility, but not the nerve, but inflammation of dentine.

The Doctor continued to discuss the primary and secondary effects of arsenic on nerves and dentine, going into the circulation, appearance and decline of inflammation, and would reassert the impossibility of cutting off a portion of the nerve.

Dr. IDDINGS asked the President, if in saying he fills to the point, he means apex?

The President answered, yes. Admits, with friend Spelman, the impossibility of cutting off a portion of the nerve, and leaving the remainder. But he who has the most practice and skill will be most successful. When difficulties present themselves, then is the time to exercise your genius and skill, and aim to come as near as possible. Be faithful to your patient and the profession. If we have used no poison we can, by care and skill, know certainly that the fang is healthy. Believes that after nerve is removed, the vitality of the tooth remains, and is supplied by the external investing membrane, and to the depth to which the supply extends. Believes that the nerve supplies vitality to the inner portion of the tooth, and in destroying the nerve we destroy vitality as far as the tooth is supplied from that source.

Dr. SLOSSON repeated his proposition. Removed the nerve with the three fangs attached, if a molar, and then is satisfied that it is removed. Nature covers and protects the living, not the dead.

Dr. ROBINSON always aims to get as near the end as possible. Would rather risk going through than have any uncertainty. Does not use a drill, but uses a piece of soft steel dressed small; crooks the end, and cleans out as he best can. He gets as near the end as possible; does not say he does it, but always aims to.

Dr. ATKINSON: This whole subject resolves itself into a truthful, honest, faithful, and persistent exercise of the functions of the operator. Step by step from inception to completion of the various processes, which chain, when no links are missing, becomes at last a perfect success. And, in proportion to the number of links there are out of the chain, we will have to hear the doleful lamentations or the exultant joy, with their modifications, all the way from blank and absolute failure consequent thereupon, to the most perfect and glorious success; and the best way to arrive at definite rules by which to be governed, is to keep a faithful record of all we do, so that by reference thereto, a reliable mode of treatment may be finally established, not forgetting to enumerate our failures as well as our successes. In the matter of filling, so much has been said that he did not want to occupy the time of the Convention. If we seal the apex and the outer part, the tooth is safe. It is pure cowardice to extract a tooth when there is a possibility of saving it. He here related the case of an inferior molar. The pulp was dead in one fang; all the rest of tooth healthy. With great care and patience it was cleansed and filled, and is now sound and serviceable. We all fail sometimes, and for himself, would say here, that he fails in more cases than he is willing to acknowledge. Uses crystal gold and adhesive foil. Vitality is supplied by the pulp, through each of the tubuli. There is knowledge enough in the profession, if every one will come forward, (either in societies, conventions, or dental periodicals,) and *spill over*, and all come forward and *gather up*. The age of patients when fang filling may be expected to be most successful, is from 15 to 45 years. Seven-tenths of the failures occur from mere shilly-shally—" I guess so." You must energise the patient with the confidence you have in the operation yourself, so that they shall deem the treatment and operation is of importance, so that they will follow instructions, and come at the proper time. When one-half or more of periostial attachments are perfect, we can expect success. When fangs are only half developed, fills with tin foil; it requires less pressure, and forms an oxide which assists to consolidate the tubuli.

Dr. SPELMAN has removed ten fillings and found the dentine very hard.

Dr. SLOSSON filled temporarily with tin; and some that he was unwilling to go out as his work, found, when removed, that the dentine was so hard that it could scarcely be cut.

Dr. STRICKLAND was aware of an ancient theory that lead in contact with living tissue, preserves the tooth. Does not believe it. Prefers to depend upon a pure and unchangeable article—gold. So far as his observation has gone, has found no reason to change. Is willing to learn and change, if shown to be wrong. Has removed tin filling, and found a black deposit, or discoloration, under it.

Dr. ATKINSON: Every thing is *pure* which has no other substance mixed with it; but even a stickler for that, has found teeth preserved with living nerve beneath, filled with the meanest kind of old crockery amalgam.

Dr. BUTLER related a case of the removal of a filling where he had filled three times. Would remove and fill a dozen times rather than leave it. Would fill with gold first, last, and all the time. First time after removing found it had gnawed down into pits; removed the second time and found again that the decay had eaten down and softened the dentine. Third time, removed only a portion and found all solid. The reason for removal was extreme sensitiveness. In children of six years, in filling molars, would use tin, because it is softest. Another case of extremely soft teeth; removed filling and cut down, but could hardly tell whether he was cutting the healthy or decayed dentine, as the tooth was so soft. In one case cut down until the nerve was touched and bled freely. Treated it with creosote, and filled without capping. The tooth is now healthy.

This occurred ten months ago—patient eleven years old. Very certain if nerve had died, there would have been serious difficulties, inflammation and discoloration.

Dr. BUTLER related some other cases in which the points of the nerve had been clipped off with excavators, which were filled and saved. Also one in which the tooth died. If an operator can satisfactorily fill and save children's teeth, he would think better of him, if he could not adults. Put in good filling every time, if you can. In imperfections of fangs in children's teeth, much care must be taken not to apply too much pressure; use small instruments.

Dr. ATKINSON called Dr. B. up for a particular purpose. Patient, strumous diathesis, a perfect blonde. Teeth large but frail; the tubuli but poorly consolidated; gold, when perfectly consolidated, is the most powerful conductor of any single metal.

Dr. Butler had to watch very closely, being extremely sensitive. In the repeated operations had discovered that each time the dentine had become more dense and solid than before.

Dr. Slosson had, in class of teeth spoken of by Dr. Butler, soft and chalky, filled with tin, for reason named by Dr. Spelman, viz: expense, and found also that they can be packed so as to exclude moisture, with less pressure than with gold. This is particularly desirable when teeth are sensitive, as a temporary protection. We have to suffer in reputation from such filling, but are willing to do so for the greater benefit of the patient.

### AFTERNOON SESSION.

Met at 2 o'clock. Discussion resumed.

### Fifth Subject—*Mechanical Dentistry.*

Dr. Atkinson remarked that to say anything that would amount to anything, would be to begin at Hippopotamus and proceed through all the various modes of mounting teeth and constructing artificial dentures, down to the new process of mounting teeth by means of amalgam, discussing all the intermediate grounds. None have tried to become adepts in all the various processes. Some are money-wise to pursue a speciality. Some denounce one process and advance another, either from want of knowledge to appreciate the one, or knowing too much to adopt another. That there is a *best* process, there is no doubt. Block teeth, properly mounted, or the late continuous gum work, is the perfection of all work. The vulcanite process comes next; then gold and silver by the ordinary process. This last is among the first, and will probably never go out of use. It is claimed by the Coralite Co. that rubber softens in oils. The Rubber Co. claim that the coralite fractures, scales, and is fragile. There are, therefore, three processes which demand attention, continuous gum, vulcanite, or coralite, and good gold work. On both sides some of the high things said must be lowered, and some of the low things elevated. A plan which has been cultivated for three years, and at one time the foolish idea of having it patented was entertained, is to take the porcelain work to form the teeth and arch, and then mount on gutta percha, vulcanized.

Dr. Robinson objected to Dr. A.'s plan, on the ground that it would be too thick and clumsy.

Dr. Atkinson exhibited some specimens of English porcelain teeth, which were much admired, having a depth of tone and appearance of vitality, rarely seen. There is great translucency of body, and yet darkly shaded.

Dr. Wilson, of Rochester, N. Y., by request made some remarks on the vulcanite base. Commenced using it with much caution. The first case was a patient who had worn a gold plate satisfactorily, and had no confidence in anything else. The plates were constructed on upper denture, and put in the mouth; the gold plates being preserved to meet emergencies in case of accident, or if the vulcanite would not answer. From time to time, patient expressed dissatisfaction, but when questioned in regard to the matter could make no objection, except that he did not believe they would last. Allowed him to wear the plate about a year, at which time the patient ordered an entire set. The Doctor, however, insisted that he should try the gold again, that he might not be deceived. He did try it, but would not take it from the office, remarking that it felt "too much like a horse-shoe."

Inserted a great many pieces since, and has experimented in all forms—full sets, and partial; suction plates and clasp: single teeth, and sectional; and the more he used it, the better he liked it. The more perfect adaptation and congeniality being important advantages. An operator with any scientific skill at all, can always be sure of a fit, the *impression being correct*, and, in consequence, the plaster model also. The fit must be absolute. Rubber clasps will last as long as gold; he would not have believed it two years ago. Does not approve of clasps—always prefers suction; but when clasps must be used, rubber is better than gold. The fit being more perfect than metal plates can be, they require but little support from clasps, only a little bracing. The Doctor here gave the history of several partial sets, inserted with clasps, which have been worn a year or more. In all he has out about 200 cases, and the more he does, the more he is satisfied of its superiority. It can be so perfectly fitted around the necks of the teeth, that it will shed water, it being light and strong. At first he was *afraid* to make it thin enough; now makes it as thin as good gold plate. Other advantages are that it can be moulded to suit any case, building out to any fullness, or making depressions where desirable. To repair a case broken by accident, or otherwise, the same process of manipulating and steaming is repeated, as far as constructing a new piece. See advertisements and circulars published.

The Doctor closed his remarks by informing the Convention that he had with him a supply of teeth and materials for the vulcanite work, with which he would be pleased to supply any who wished to purchase. He would also take orders for vulcanizing machines and office rights, and would be happy in answering questions from any one present.

Dr. Butler said he had used both rubber and coralite, and had cut them to pieces to remove the teeth, so as to repeat experiments. The difference between the two materials scarcely perceptible, except in color. A few days ago a hole occurred by accident in a piece of coralite. The Doctor wishing to remove the teeth to remodel the case, cut it to pieces with a great effort; it was almost impossible to cut it out around the teeth and pins. The union was so close, it formed a *strong adhesion*—mechanical, of course.

Dr. Strickland tried some experiments. Had been told that nitro-muriatic acid would dissolve it. Put a plate of vulcanite into a vessel with the acid, and found that it gradually diminished. Removed it next day, and renewed the acid, and observed it still diminishing. Again renewed the acid and allowed it to remain several hours, when it parted along the meridian line. He then placed it in a dish with the acid over a spirit lamp, and found that it dissolved in a few minutes

SIXTH SUBJECT—*Miscellaneous.*

Dr. BUTLER would like to show those who are incredulous on the subject of fang filling, a decided success, in a young man's mouth here present. A case was here examined, in which an upper molar had been under treatment, and now two of the fangs are filled, and the other excavated ready for filling; all sound, clean, and clear, not the least sign of inflammation. The examination was made with much satisfaction, and seemed to satisfy all.

Dr. GYLE exhibited a tooth of which the fang and crown had been filled. It had been worn in the mouth with great satisfaction, when the patient took cold and a slight inflammation set in. In the meantime some personal misunderstanding had occurred between the patient and the operator, who concluded to "bite off his nose to spite his face," by steadily declining to call on the operator for relief, preferring to permit the inflammation to increase until he could endure the pain no longer; then went to another dentist and had it extracted, when a sack was discovered on the end of each fang. The tooth was broken open and exhibited most *excellent* workmanship.

Some spicy remarks were made, but being of a local and personal nature, we do not report them.—REPORTER.

Committee on Constitution was called, and report accepted. The constitution of the Indiana Association, as published in the March number of the *Dental Register*, was read.

Dr. SPELMAN motioned that we organize temporarily under the Indiana Constitution, with such changes in language as to suit this society, proceed to elect officers and appoint committees to revise the constitution, and present at next meeting. Carried.

Dr. ATKINSON made a motion that the present officers be appointed charter members and an examing committee for the election of future members of this Association. Adopted.

Dr. ROBINSON motioned that we adjourn to meet here again on the second Tuesday in May, 1860. Carried.

The officers of the present Convention elected to their assistance the following gentlemen, viz: Drs. W. B. Ingersoll, W. P. Horton, B. F. Spelman, C. Palmer, E. J. Way, and J. F. Siddell, to form a constitution, organize and found a local association.

After which, at the invitation of the resident dentists of Cleveland, all adjourned to Mr. Stacey's, where a most sumptuous table was awaiting them, and the evening was passed very pleasantly by all parties.

The charter members will form a constitution and by-laws and give due notice when they are ready for applications for examinations from those wishing to become members.—REP.

## Figures and Tables

**Fig. 1 f1:**
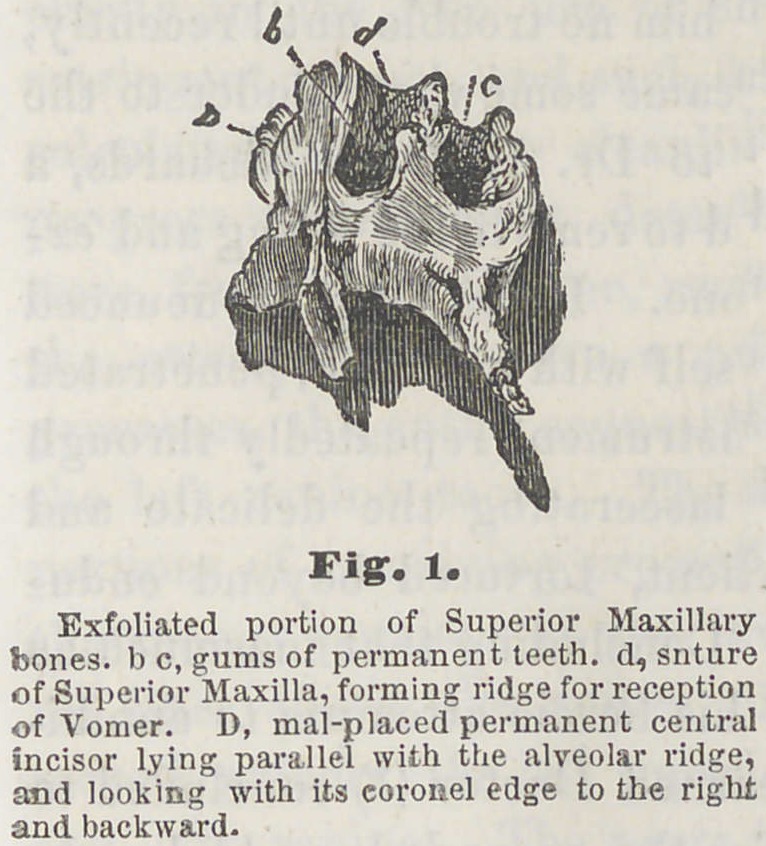


**Fig. 2 f2:**
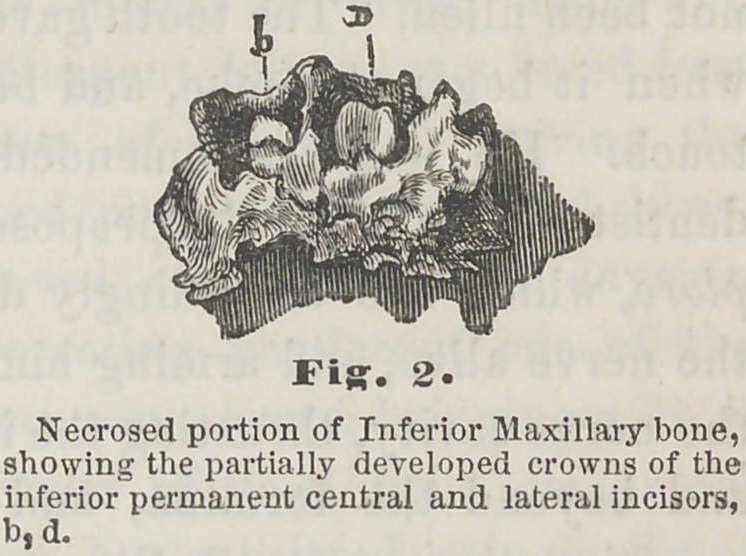


**Fig. 3 f3:**